# Fraternal Journalism

**Published:** 1881-04

**Authors:** 


FRATERNAL JOURNALISM.—We are deeply indebted to our esteemed confrere of the *Maryland Medical Journal* for the kind and complimentary manner in which he has been pleased to speak of our *Popular Science* department, and the generally complimentary words he uses in regard to our enterprise. Our relations with our neighbor, cordial in the beginning, have grown with our growth, and strengthened with our strength; and to-day there is no more welcome visitor to our editorial sanctum than the *Journal*, over which he so pleasantly presides. We wish him, and his *Journal* success, commensurate with his industry and efforts to please his readers.

